# A Japanese Prospective, Multicenter Study of Colonic Stenting for Palliation Using a High Axial Force Self-Expandable Metal Stent for Malignant Large Bowel Obstruction in 200 Patients

**DOI:** 10.3390/jcm12155134

**Published:** 2023-08-05

**Authors:** Rei Ishibashi, Takashi Sasaki, Hiroyuki Isayama, Takeaki Matsuzawa, Toshio Kuwai, Tomonori Yamada, Shuji Saito, Masafumi Tomita, Toshiyasu Shiratori, Satoshi Ikeda, Hideki Kanazawa, Mitsuhiro Fujishiro, Iruru Maetani, Yoshihisa Saida

**Affiliations:** 1 Department of Gastroenterology, Graduate School of Medicine, The University of Tokyo, Tokyo 113-8655, Japan; a4mb1011-thk@umin.ac.jp (R.I.); fujishiromi-int@h.u-tokyo.ac.jp (M.F.); 2 Department of Hepato-Biliary-Pancreatic Medicine, Cancer Institute Hospital, Japanese Foundation for Cancer Research, Tokyo 135-8550, Japan; sasakit-tky@umin.ac.jp; 3 Department of Gastroenterology, Graduate School of Medicine, Juntendo University, Tokyo 113-8431, Japan; 4 Department of Surgery, Imusumiyoshi General Hospital, Saitama 354-0041, Japan; matsuzawatakeaki@yahoo.co.jp; 5 Department of Gastroenterology, National Hospital Organization, Kure Medical Center and Chugoku Cancer Center, Hiroshima 737-0023, Japan; kuwai.toshio.ur@mail.hosp.go.jp; 6 Department of Gastroenterology, Japanese Red Cross Aichi Medical Center Nagoya Daini Hospital, Aichi 466-8650, Japan; tohiryuhi@gmail.com; 7 Department of Surgery, Gastrointestinal Center, Yokohama Shin-Midori General Hospital, Kanagawa 226-0025, Japan; shusaito@jb3.so-net.ne.jp; 8 Department of Surgery, Kobe Tokushukai Hospital, Hyogo 655-0017, Japan; naturaltomato@gmail.com; 9 Department of Gastroenterology, Kameda Medical Center, Chiba 296-8602, Japan; t_shiratori0126@yahoo.co.jp; 10 Department of Gastroenterological Surgery, Hiroshima Prefectural Hospital, Hiroshima 734-8530, Japan; sikeda1965@gmail.com; 11 Department of Surgery, National Hospital Organization Sagamihara Hospital, Kanagawa 252-0392, Japan; kanazawa.hideki.ma@mail.hosp.go.jp; 12 Department of Gastroenterology, Sin-Kuki General Hospital, Saitama 346-8530, Japan; mtnir50637@med.toho-u.ac.jp; 13 Department of Surgery, Toho University Ohashi Medical Center, Tokyo 153-8515, Japan; yoshisaida@nifty.com

**Keywords:** axial force, chemotherapy, malignant colorectal obstruction, palliation, self-expandable metal stent

## Abstract

Evidence of the efficacy and safety of colorectal stent placement for palliation remains insufficient. This single-arm, prospective, multicenter study with a WallFlex enteral colonic stent included 200 consecutive patients with malignant large bowl obstruction in the palliation cohort. The technical and clinical success, as well as stent patency and complications as short-term (≤7 days) and long-term (>7 days) outcomes, of high axial force self-expandable metal stent (SEMS) placement was evaluated. The technical and clinical success rates were 98.5% and 94.5%, respectively. Non-recurrent colorectal obstruction at 1 year was 63.9%, and 71.2% of the patients remained free of recurrent colorectal obstruction until death or the last follow-up. Fifty-six patients (28.0%) received chemotherapy, and five patients were administered bevacizumab after stent placement. The overall complication rate was 47%, including four (2.0%) early-onset and ten (5.0%) late-onset perforations, mostly due to stent-edge injury. Only the use of a long SEMS was a risk factor for perforation. In conclusion, endoscopic colorectal stenting using high axial force SEMS is an effective and safe procedure for palliation in patients with malignant colorectal obstruction. However, care should be taken to avoid perforation at the stent edge when using a long SEMS.

## 1. Introduction

Advanced cancers sometimes result in malignant colorectal obstruction [1], which may be caused by the colorectal cancer itself, tumor invasion of other organ cancers, or peritoneal dissemination. Although surgical resection of the obstructed colon and anastomosis with an intact colon or surgical bypass is ideal, surgery is often difficult, especially when the cancer is at an advanced stage. In such cases, colostomy or endoscopic colorectal stenting is an alternative treatment option. However, there are reports that ostomy influences quality of life negatively [2,3,4], and endoscopic colorectal stenting for palliation (PAL) is therefore favored.

Endoscopic colorectal stenting for PAL still faces various challenges. First, there are many extracolonic stenoses, and limited data exist regarding the short-term efficacy and safety of stenting [5,6,7,8,9,10]. In addition, data on the long-term outcomes are limited. There is also insufficient evidence regarding the safety of stenting in combination with drug therapy [11,12,13]. Data on outcomes associated with different stents are also limited [14,15,16], and numerous data are integrated with bridge-to-surgery (BTS) cases, with limited data available for PAL only [17,18].

The Japan colonic stent safe procedure research group (JCSSPRG) was launched in 2012 to promote the safe use of colonic stents. We conducted several multicenter prospective studies to investigate the safety and efficacy of colonic stenting using various self-expandable metal stents (SEMSs). The WallFlex enteral colonic stent was the first SEMS approved by public insurance for endoscopic colonic stenting in Japan, and we conducted a multicenter study to evaluate its safety and efficacy. The study included both BTS and PAL cohorts. We previously reported the short-term outcomes of the entire cohort and the short- and long-term outcomes of the BTS cohort [9,15,16]. We have accumulated data from the early days of stent introduction in Japan, and, here, we report the short- and long-term outcomes of the PAL cohort.

## 2. Materials and Methods

### 2.1. Study Design

This single-arm, prospective, multicenter study by the JCSSPRG evaluated the efficacy and safety of colorectal stenting using an uncovered WallFlex enteral colonic stent. Fourteen academic centers and 32 community hospitals participated in the study. Institutional review board approval was obtained for patient enrollment at each institution prior to the start of the study. Each patient provided consent to undergo the procedure and registered for the study. This study was registered in the University Hospital Medical Information Network Clinical Trial Registry (UMIN00007953). 

Patients with malignant large bowel obstruction were included in this study. Large bowel obstruction was diagnosed using abdominal radiography, colonoscopy, or computed tomography. Patients with a history of colonic stenting were excluded. Other exclusion criteria included enteral ischemia, suspected or impending perforation, intra-abdominal abscess, severe inflammatory changes around the obstruction, and contraindication to endoscopic treatment.

Each patient was registered before or immediately after SEMS placement. All clinical data were collected prospectively. At that time, patients who were scheduled for surgical resection were classified as BTS, and patients who were not scheduled for surgical resection were classified as PAL. We selected only the PAL cohort and evaluated short- and long-term efficacy and safety of colonic stenting using the WallFlex enteral colonic stent.

### 2.2. Endoscopic Stent Placement

Stent placement was performed via colonoscopy under fluoroscopic guidance. Before starting this prospective study, we had developed the technical guidelines for safe colonic stenting (“the JCSSPRG Mini-Guidelines” [19]) and tried to share tips for stent placement and points to avoid complication with the group members. Briefly, the colonoscope was gently inserted into the obstruction site, the catheter and guidewire were manipulated to pass through the stenosis, contrast medium was injected to identify the stenosis and measure the length of the obstruction site fluoroscopically, and, finally, the stent was deployed across the stenosis under endoscopic and fluoroscopic views using the through-the-scope method. Intraluminal marking with a clip was recommended to identify stricture location. Stricture dilation was avoided to prevent perforation. SEMS placement was performed or supervised by an endoscopy expert from the JCSSPRG. Only uncovered WallFlex enteral colonic stents (Boston Scientific Corporation, Natick, MA, USA), 22 and 25 mm in diameter and 6, 9, or 12 cm in length folded in 10 Fr. delivery system, were evaluated in this study.

### 2.3. Definition of Outcomes

The colorectal obstructing scoring system (CROSS) was constructed by JCSSPRG based on a scoring system for malignant gastric outlet obstruction [20,21]. CROSS evaluates oral intake as follows: CROSS 0, requiring continuous decompression; CROSS 1, no oral intake; CROSS 2, liquid or enteral nutrient intake; CROSS 3, soft solids, low-residue, and full diet with symptoms of stricture; CROSS 4, soft solids, low-residue, and full diet without symptoms of stricture.

Technical success was defined as successful deployment of the stent across the entire length of the stricture. Clinical success was defined as the resolution of symptoms and radiological relief of the obstruction within 24 h, as confirmed by radiographic observation [14].

Complications after SEMS placement included perforation, stent migration, recurrent colorectal obstruction (RCRO), bleeding, infection or fever, abdominal pain, tenesmus, fecal incontinence, and other minor complications. Bleeding that did not require treatment was defined as minor bleeding, whereas bleeding requiring treatment was defined as major bleeding. Early-onset was defined as occurring within 7 days and late-onset as occurring after 7 days. The incidence rate was calculated using the denominator of 200 patients enrolled in the PAL cohort.

### 2.4. Data Collection and Statistical Analysis

All clinical data were prospectively reported using an electronic registration system. At enrollment, patient characteristics—including age, sex, Eastern cooperative oncology group (ECOG) performance status (PS), etiology, CROSS, and symptoms of obstruction—were recorded. The obstruction number, type of obstruction, stricture length, and location of the obstruction were collected as tumor characteristics. The number of stent placements, length and diameter of the stent, balloon dilation before stent placement, procedure time, technical and clinical success, and causes of clinical failure were evaluated as the outcomes. Complications were investigated to assess safety, and the influence of concomitant chemotherapy was evaluated.

Categorical variables are expressed as absolute numbers and percentages. Continuous variables are presented as medians and ranges. Overall survival and time-to-RCRO (TRCRO) were calculated using the Kaplan–Meier method. Risk factors for perforation were evaluated using the chi-square test and a logistic regression analysis model, which included the following variables: age, sex, etiology, type of obstruction, length of stent, and chemotherapy after SEMS placement. All statistical analyses were performed using JMP Pro software (version 16.0; SAS Institute, Chicago, IL, USA).

## 3. Results

### 3.1. Patient Characteristics

A total of 517 patients were enrolled in this study from March 2012 to October 2013. After excluding ineligible and BTS cases, 200 patients were included in the PAL cohort. The PAL cohort patient characteristics are shown in Table 1. The median age was 74.5 years, and 55.5% of the patients were male. Sixty percent of the patients had ECOG PS 0 or 1, while the remaining 40% had ECOG PS 2 or higher. The most common etiology was colorectal cancer (72.5%), followed by gastric (15.5%) and pancreatic cancer (5.5%). Most patients (97.5%) had symptoms of obstruction. Only 39.0% had a CROSS of 0.

The details of the obstruction sites are summarized in Table 2. Four cases exhibited two obstruction sites. Therefore, 204 obstruction sites were managed using colorectal stenting. Extracolonic obstruction accounted for 31.5% of the obstructions. There were 54 (27.0%) patients with stenosis with peritoneal dissemination. Median obstruction length was 4.0 cm. Most obstructions were located on the left side of the colon (69.6%), with sigmoid colon stenting being the most common.

### 3.2. Short-Term Outcomes of Stent Placement

Digestive tract decompression prior to stent placement was performed in 58 (29%) patients via nasogastric (n = 14), nasointestinal (n = 16), and transanal (n = 28) tube insertion. Cleansing enemas and oral bowel cleansing were performed as preparation for stent placement in 70 (35.0%) and 10 (5.0%) patients, respectively. Obstruction sites were marked with intraluminal clipping and extracorporeal marking in 107 (53.5%) and 19 (9.5%) patients, respectively.

The short-term outcomes are shown in Table 3. Most patients were treated with a single stent (95.9%). The main stent type was 6 cm in length and 22 mm in diameter. Balloon dilation before stent placement was performed in only one patient (0.5%). The median procedure time was 30 min (range; 6–170).

The technical and clinical success rates were 98.5% and 94.5%, respectively. Technical failures resulted from failure of guidewire cannulation (n = 2) and perforation by a catheter (n = 1). Among the patients with technical success, clinical failure occurred in only eight cases, due to insufficient stent expansion (n = 3), stent kinking (n = 1), stent migration (n = 1), perforation by stent injury (n = 1), perforation due to delayed decompression (n = 1), and proximal small bowel obstruction (n = 1). 

Early-onset (≤7 days) complications occurred in 28 (14.0%) patients and are shown in Table 4. There were four (2.0%) cases of perforation, of which one case was perforated during the procedure and did not achieve technical success, two cases were perforated within 24 h and did not achieve clinical success, and the remaining case was perforated by stent-edge injury 4 days after stent placement. Of these, two patients underwent surgery: one underwent primary resection and colostomy, the other underwent stoma construction, and both recovered after surgery. The other two patients had poor prognoses and were treated conservatively but died. Perforations were caused by the catheter during procedure (n = 1), stent-edge injury (n = 2), and delayed decompression (n = 1). There were two cases of perforation involving the stent and its surroundings. Stent migration occurred in one case, and the stent was discharged from the anus. Other minor complications, such as bleeding (n = 4), infection or fever (n = 8), and abdominal pain (n = 9), improved upon conservative treatment.

### 3.3. Long-Term Outcomes of Stent Placement

**Figure 1:**
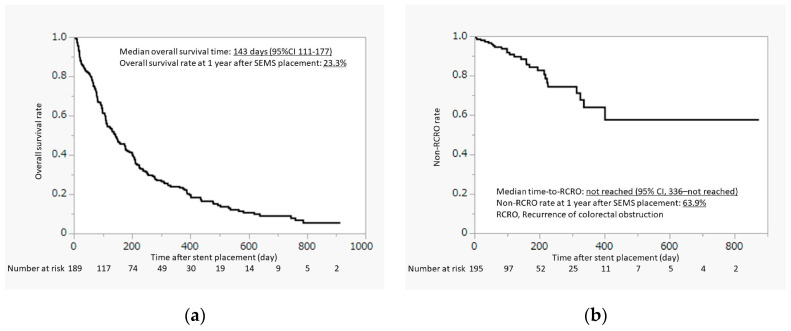
Overall survival and time-to-recurrent colorectal obstruction after stent placement. (**a**) Overall survival after stent placement; (**b**) Time-to-recurrent colorectal obstruction after stent placement.

Overall survival and TRCRO after stent placement are shown in Figure 1. The median overall survival was 143 days (95% confidence interval [CI], 111–177 days) and the overall survival rate at 1 year was 23.3%. The median duration of TRCRO was not reached (95% CI, 336–not reached), and the non-RCRO rate at 1 year was 63.9%. Most patients (71.2%) remained free of RCRO until death or the last follow-up.

Late-onset (>7 days) complications occurred in 66 (33.0%) patients and are shown in Table 4. There were 10 (5.0%) cases of perforation. Only two patients underwent surgery, and the other patients were considered to have a poor prognosis and were treated conservatively. Although most perforations were thought to be related to the stent and its surroundings (n = 5), there were cases with unclear causes (n = 4) or perforation of the tumor at another site (n = 1). Stent obstruction occurred in 24 (12.0%) patients, of which four were treated surgically, nine underwent additional stenting, six underwent stent cleaning, one underwent ablation with argon plasma coagulation (APC), and four received conservative treatment. Regarding the surgical procedures, all four patients underwent colostomy and were found to be alive for more than two months. Proximal gastrointestinal obstruction was observed in 12 (6%) patients. 

Fifty-six (28.0%) patients underwent chemotherapy after stent placement. There were 38 (26.2%) cases of 145 colorectal cancer cases, 15 (48.4%) cases of 31 gastric cancer cases, and one case each of cervical, ovarian, and pancreatic cancer (Table 5). For colorectal cancer, oxaliplatin-based therapy was administered to 20 patients and irinotecan-based therapy was administered to 12 patients. Nine patients opted for the relatively less invasive oxaliplatin- and irinotecan-free chemotherapy, as follows S-1, capecitabine, UFT + leucovorin, trifluridine/tipiracil, and cetuximab. Bevacizumab, an anti-vascular endothelial growth factor (VEGF) monoclonal antibody, was administered in five (13.2%) cases. Panitumumab or cetuximab, anti-epidermal growth factor receptor (EGFR) antibodies, was used in 13 (34.2%) cases. Stent migration was observed in 5 (13.2%) of the 38 chemotherapy cases, 2 of which received molecular targeted drugs (panitumumab and bevacizumab). Perforation was observed in two (3.6%) patients, both with colon cancer. One received 5-fluorouracil plus folinic acid plus irinotecan (FOLFIRI) with bevacizumab until just before perforation, which occurred 145 days after stent placement. The patient was managed surgically. Another patient received 5-fluorouracil plus folinic acid plus oxaliplatin (FOLFOX), and perforation occurred 85 days after stent placement. The patient died nine days after the perforation.

### 3.4. Risk Factors for Perforation

Univariate analysis was performed on risk factors for perforation (Table 6). Only the use of a long SEMS was a risk factor for perforation. Compared with 6 cm SEMS, the hazard ratio of 12 cm SEMS was 7.37 (95% CI, 1.53–35.58; *p* = 0.03), indicating that the 12 cm SEMS was associated with perforation. The use of chemotherapy, including anti-VEGF antibody drugs, was also not a risk factor.

## 4. Discussion

This study investigated the short- and long-term efficacy and safety of SEMS placement of a WallFlex enteral colonic stent for palliation. This study is data on PAL at the time of the introduction of colorectal stenting to Japan, and data on long-term prognosis of a large number of patients are also analyzed. The technical and clinical success rates were very high (98.5% and 94.5%, respectively) in the PAL cohort, and more than 70% of patients remained free from RCRO until death or the last follow-up. Stenting for PAL was beneficial even in patients with poor general condition or prognosis, with 28.0% of patients receiving chemotherapy, a one-year survival rate of 23.3%, and a 63.9% non-RCRO rate at one year. The cumulative perforation rate was 7%, with perforation during the procedure in one case, within 7 days after SEMS placement in three cases, and longer than 7 days after SEMS placement in ten cases. Only the use of a long SEMS was a risk factor for perforation.

The WallFlex enteral colonic stent was woven using only a cross-nitinol wire, which provides WallFlex with a high axial force, and an axial force zero border with a very small angle, which provides a sustained pressure load on the intestinal wall [22]. We previously reported the short-term efficacy and safety of colonic stenting with WallFlex in BTS and the long-term outcomes after BTS [15,16]. In this study, we found that short-term placement of WallFlex for colorectal cancer is safe and has minimal oncological impact.

Previous studies for PAL are summarized in Table 7. As the definition of clinical success varied between papers, the total clinical success rate was calculated using the number of patients with malignant large bowel obstruction as the denominator. Unfortunately, many previous reports do not distinguish between short- and long-term complications. One report showed comparable efficacy and safety for the WallFlex and Niti-S D-type, which was woven as hooks and cross stents [23]. However, other studies using WallFlex reported a high perforation rate that was attributed to the structure or large diameter (including stents with a 25/30 mm in diameter) of the stent [24,25]. The cumulative perforation rate in this study was 7%, which was comparable to other studies. As shown in Table 7, previous studies reported higher perforation rates for WallFlex than for Niti-S D-type stents [14,23,24,26,27]. The short-term results of this study are comparable with those reported previously. Although the perforation rate with WallFlex in the PAL cohort may be comparable, it is unclear whether there are differences in perforation rates between stents, as there are few reports of long-term placement of each stent.

Previous reports of endoscopic colorectal stenting for PAL tend to have lower clinical success rates (54.1–96.0%) than those for BTS because of numerous extracolonic obstructions [10,28,31,32]. Furthermore, peritoneal carcinomatosis is technically difficult, which lowers the technical success rate of PAL compared with that of BTS [36]. A study limited to colorectal obstruction from gastric cancer reported technical success of 73.9% and clinical success of 54.1%, which is very low compared with that of primary colorectal cancer [31]. In the present study, the technical and clinical success rates of extracolonic obstruction were 98.2% and 91.1%, which compare favorably with previous reports [27,29,31,32]. The number of extracolonic cases in this study was 63 (31.5%), a smaller proportion than in previous reports. This may also be a reason for the good clinical success.

The WallFlex colonic stent has a strong radial force, which may have helped maintain the stent lumen, contributing to its good outcomes in the present study [22]. Evaluation of complication rates revealed that the previous reports for PAL had a higher perforation rate (7.9–8.9%) than those for BTS because of the long-term placement of the stent [9,17,28]. In PAL cases, unlike BTS, perforation due to disease progression does not always result in surgery, and it is sometimes difficult to identify the site of perforation and whether the perforation was caused by the stent. In this study, there were seven cases of perforation involving the stent and its surroundings. Age ≥70 years and sigmoid colonic location were found to be independently associated with the occurrence of early perforation, and stent location in the flexure and absence of peritoneal carcinomatosis were significantly associated with delayed perforation [24]. The three cases of early perforation in this study were among patients aged over 70 years, and three cases exhibited sigmoid colon or rectosigmoid obstruction, which corresponds to previous reports. Of the late perforations seen in the present study, five patients had peritoneal dissemination, and only one patient had a stent placed in flexion. Stenting in flexure or the sigmoid colon is a risk factor for perforation due to the increased risk of stent-edge injury. A univariate analysis of perforation showed that a 12 cm long stent is a risk factor because it may be more prone to edge injury. However, the colon has many fixed and unfixed areas and is subject to peristalsis and intestinal floating, which makes it difficult to predict the associated risk of stent placement. In extrinsic cases, the intestine may become fixed, and care must therefore be taken when placing the stent, especially at bends. 

The relationship between SEMS placement and overall survival could not be evaluated as this cohort included patients with different cancer origins. However, the median overall survival and TRCRO were both good, and 121 of 170 patients (71.2%) were free of RCRO until death or the last follow-up. SEMS placement could therefore improve quality of life, eliminating the need for additional surgery, and allow recovery from large bowel obstruction. Even in cases of RCRO, some cases can be treated with additional stents, APC ablation, and stent cleaning.

Fifty-six (28.0%) patients underwent chemotherapy after stent placement. Despite the limited number of adverse events in the present study, chemotherapy was not a risk factor for perforation. Some reports indicate that stenting during and after chemotherapy is safe and effective, whereas others contraindicate stenting [37,38]. Furthermore, bevacizumab has been associated with higher complication rates, nearly tripling the risk of perforation [30,35]. In the present study, we observed one case of perforation on FOLFIRI + bevacizumab. In this case, the normal mucosa at the edge of the stent perforated. The perforation may have occurred because of delayed wound healing caused by angiogenesis inhibitors. According to previous reports, the perforation rate increased to 15.4% with bevacizumab, and the median time to perforation was 21 days [30,35]. Perforations involving bevacizumab may be occurring late-onset. Considering the functional mechanism of anti-VEGF antibody drugs and the hazard ratio in this study, it is possible that anti-VEGF antibody drugs may be involved in perforation. The European Society of Gastrointestinal Endoscopy guidelines recommend that antiangiogenic therapy can be considered in patients following colonic stenting, but that colonic stenting should be avoided during antiangiogenic therapy [39]. As acute colorectal obstruction and perforation are potentially fatal, either surgery or stenting is required, even during systemic chemotherapy including bevacizumab therapy. Further studies are therefore needed to elucidate the relationship between SEMS placement and chemotherapy and, in particular, stenting during treatment with anti-VEGF antibody drugs.

In recent years, hook- and cross-type stents, which have lower axial and radial forces, have been widely used for colorectal stenting. Hook-type SEMSs fold when longitudinal force is applied to the edge of the stent, according to the hook structure. In contrast, cross-type stents are not folded and may increase the risk of stent tip or edge injury. Care must therefore be taken in stent selection, because the flared portion of a stent often has a cross structure.

This study had several limitations. Although this study prospectively evaluated a large number of patients compared with previous reports, it was a single-arm observational study. Future studies should include a prospective, randomized controlled trial comparing WallFlex enteral colonic stents with other stent types, such as hook and cross stents. Furthermore, clinical success and TRCRO were regarded as positive outcomes. However, these outcomes may be insufficient to judge the impact of the stenting on these patients, and a patient questionnaire should be used to assess quality of life. Finally, although the administration of chemotherapy and anti-VEGF antibody drugs after stenting were investigated, the influence of these therapies prior to stenting was not evaluated, and the relationship between chemotherapy and perforation requires further elucidation.

## 5. Conclusions

This large, multicenter, prospective study demonstrated the efficacy and safety of palliative stenting with high axial force SEMSs for malignant colorectal obstruction. Most patients did not require additional surgery and remained free of RCRO until death or the last follow-up. However, care should be taken to avoid perforation at the stent edge with long-term stent placement when using a long SEMS.

## Figures and Tables

**Table 1:** Patient characteristics (n = 200).

**Age, Years, Median (Range)**	**74.5 (63–85)**
Sex, male, n (%)	111 (55.5)
ECOG performance status, n (%)	
0	22 (11.0)
1	98 (49.0)
2	32 (16.0)
3	34 (17.0)
4	14 (7.0)
Etiology, n (%)	
Colorectal cancer	145 (72.5)
Gastric cancer	31 (15.5)
Pancreatic cancer	11 (5.5)
Others ^†^	13 (6.5)
CROSS, n (%)	
0	68 (39.0)
1	58 (29.0)
2	31 (15.5)
3	35 (17.5)
4	8 (4.0)
Symptoms of obstruction, n (%)	195 (97.5)
Deterioration of defecatory patterns	184 (92.0)
Bloating	161 (80.5)
Abdominal pain	146 (73.0)
Nausea/vomiting	95 (47.5)

n, number; ECOG, Eastern cooperative oncology group; CROSS, Colorectal obstruction scoring system. ^†^ Ovarian cancer (n = 4), cervical cancer (n = 3), uterine cancer (n = 2), esophageal cancer (n = 1), biliary tract cancer (n = 1), gallbladder cancer (n = 1), ampullary cancer (n = 1).

**Table 2:** Details of obstruction site (n = 204).

Numbers of obstruction, n (%) ^†^	
1	196 (98.0)
2	4 (2.0)
Types of obstruction, n (%) ^†^	
Colonic	137 (68.5)
Extracolonic	63 (31.5)
Peritoneal dissemination, n (%) ^†^	54 (27.0)
Length of obstruction, cm, median (range)	4.0 (3–6)
Location of obstruction, n (%)	
Left side/Right side	142 (69.6)/62 (30.4)
Rectum	26 (12.7)
Rectosigmoid junction	30 (14.7)
Sigmoid colon	49 (24.0)
Sigmoid-descending colon junction	15 (7.4)
Descending colon	22 (10.8)
Splenic flexure	19 (9.3)
Transverse colon	28 (13.7)
Hepatic flexure	5 (2.5)
Ascending colon	8 (3.9)
Cecum	1 (0.5)
Ileocecal junction	1 (0.5)

n, number. ^†^ The total number of cases was 200.

**Table 3:** Outcomes of stent placement (n = 200).

Numbers of stent placement, n (%)	
Single stent	190 (95.9%)
Double stents	7 (3.5)
No stent placement (technical failure)	3 (1.5)
Length of the stent, n (%) ^†^	
6 cm	108 (52.9)
9 cm	73 (35.8)
12 cm	23 (11.3)
Diameter of the stent, n (%) ^†^	
22 mm	183 (89.7)
25 mm	21 (10.3)
Balloon dilation before stent placement, n (%)	1 (0.5)
Procedure time, min, median (range)	30 (6–170)
Technical success, n (%)	197 (98.5)
Clinical success, n (%)	189 (94.5)
Cause of clinical failure, n (%)	
Insufficient stent expansion	3 (1.5)
Stent kinking	1 (0.5)
Stent migration	1 (0.5)
Proximal-bowel perforation	1 (0.5)
Perforation due to obstructive colitis	1 (0.5)
Proximal small bowel obstruction	1 (0.5)

n, number. ^†^ Total number of stents was 204.

**Table 4:** Complications.

	Early Onset (≤7 days) (n = 200)	Late Onset (>7 days) (n = 200)
Total, n (%)	28 (14.0)	66 (33.0)
Perforation	4 (2.0)	10 (5.0)
During the endoscopic procedure	1 (0.5)	0
At stent and its surroundings after stent placement	2 (1.0)	5 (2.5)
At other sites after stent placement	1 (1.0)	1 (0.5)
Unknown perforation sites after stent placement	0	4 (2.0)
Stent migration	1 (0.5)	9 (4.5)
Stent obstruction	5 (2.5)	24 (12.0)
Major bleeding	0 (0)	1 (0.5)
Minor bleeding	3 (1.5)	2 (1.0)
Insufficient stent expansion	1 (0.5)	0 (0)
Infection/fever	6 (3.0)	4 (2.0)
Abdominal pain	8 (4.0)	4 (2.0)
Tenesmus	3 (1.5)	1 (0.5)
Fecal incontinence	0 (0)	1 (0.5)
Ileus associated with poor peristalsis	1 (0.5)	0 (0)
Vomiting without obstruction	0 (0)	2 (1.0)
Gastrointestinal obstruction at proximal site	3 (1.5)	12 (6.0)
Interstitial pneumonitis	0 (0)	1 (0.5)

n, number.

**Table 5:** Chemotherapy regimens after stent placement.

	Regimen	N
Colorectal cancer	S-1	3
	Capecitabine	1
	UFT + Leucovorin	2
	Trifluridine/Tipiracil	1
	FOLFIRI	3
	FOLFOX	5
	SOX	1
	CAPOX	9
(anti-VEGF)	5-fluorouracil + Leucovorin + Bevacizumab	1
	FOLFIRI + Bevacizumab	2
	CAPIRI + Bevacizumab	1
	CAPOX + Bevacizumab	1
(anti-EGFR)	Cetuximab	1
	Panitumumab	1
	Irinotecan + Cetuximab	4
	Irinotecan + Panitumumab	1
	FOLFIRI + Panitumumab	1
	FOLFOX + Panitumumab	5
Gastric cancer	S-1	2
	Irinotecan	2
	Docetaxel	1
	Nab-Paclitaxel	3
	Paclitaxel	5
	Cisplatin + Irinotecan	2
	5-fluorouracil + Methotrexate	2
	S-1 + Cisplatin	1
	S-1 + Irinotecan	1
	S-1 + Docetaxel	1
	S-1 + iv. Paclitaxel + ip. Paclitaxel	2
	SOX + ip. Paclitaxel	1
Pancreatic cancer	S-1	1
Ovarian cancer	Carboplatin + Paclitaxel	1
	SOX + ip. Paclitaxel	1

N, number; VEGF, vascular endothelial growth factor; EGFR, epidermal growth factor receptor; FOLFIRI, 5-fluorouracil plus leucovorin plus irinotecan; FOLFOX, 5-fluorouracil plus leucovorin plus oxaliplatin; SOX, S-1 plus oxaliplatin; CAPOX, capecitabine plus oxaliplatin; CAPIRI, capecitabine plus irinotecan; iv., intra-venous; ip., intra-peritoneal.

**Table 6:** Factors associated with perforation.

Variables		Univariate Analysis	
	N	Hazard Ratio (95%CI)	*p* Value
Age ≥ 70	73	1.04 (0.33–3.22)	0.95
Age < 70	127	1	
Male	111	2.10 (0.64–6.94)	0.21
Female	89	1	
Etiology			
Colorectal cancer	145	0.66 (0.21–2.07)	0.48
Non-colorectal cancer	55	1	
Types of obstruction			
Colonic	137	0.59 (0.20–1.78)	0.35
Extracolonic	63	1	
Location ^†^			
Left	142	1.10 (0.33–3.68)	0.87
Right	62	1	
Length of stent ^†^			**0.03**
6 cm	108	1	
9 cm	73	3.13 (0.76–12.96)	0.11
12 cm	23	7.37 (1.53–35.58)	**0.01**
Chemotherapy after SEMS placement (+)	56	0.41 (0.09–1.88)	0.25
Chemotherapy after SEMS placement (−)	144	1	
Anti-VEGF antibody drug (+)	5	3.50 (0.36–33.62)	0.28
Anti-VEGF antibody drug (−)	195	1	
Anti-EGFR antibody drug (+)	13	-	-

N, number; CI, confidence interval; SEMS, self-expandable metal stent; VEGF, vascular endothelial growth factor; EGFR, epidermal growth factor receptor. ^†^ Total numbers of obstruction and stent were 204. Bold text indicates a statistically significant correlation with a *p*-value less than 0.05.

**Table 7:** Previous studies of SEMS placement for palliation.

Study	Year	Country	Design	Stent	N	Technical Success	Clinical Success	Perforation N (%)
van Hooft JE [24]	2008	Netherlands	Prospective	WallFlex	10	90%	90%	6 (60.0)
Kim JH [26]	2011	Korea	Retrospective	WallFlex	108	89%	86.1%	6 (6.3)
Meisner S [27]	2012	Denmark	Prospective	WallFlex	255	91.3%	76.9%	13 (5.1)
Cheung DY [23]	2012	Korea	Prospective	WallFlex	28	100%	100%	1 (3.6)
Present study	2023	Japan	Prospective	WallFlex	200	98.5%	94.5%	14 (7.0)
Total					601	96.7%	85.7%	40 (6.7)
Cheung DY [23]	2012	Korea	Prospective	D-type	30	100%	93.3%	0 (0)
Yoshida S [14]	2013	Japan	Prospective	D-type	33	100%	97%	0 (0)
Total					63	100%	95.2%	0 (0)
Sausa M [28]	2017	Portugal	Retrospective	Hanaro	45	97.8%	96.5%	4 (8.9)
Franz S [29]	2018	USA	Retrospective	Wallstent	187	76%	54.5%	7 (3.7)
Small AJ [30]	2010	USA	Retrospective	Various	168	96.0%	95.8%	15 (13.3)
Kim BK [31]	2012	Korea	Retrospective	Various	111	73.9%	54.1%	8 (7.9)
Kim BC [17]	2012	Korea	Retrospective	Various	102	87.0%	77.8%	3 (2.9)
Moon SJ [32]	2014	Korea	Retrospective	Various	97	95.9%	81.4%	2 (2.1)
Van den Berg [33]	2015	Netherlands	Prospective	Various	48	91.6%	87.5%	8 (16.7)
Kwon SJ [34]	2021	Korea	Retrospective	Various	495	92.9%	83.5%	19 (3.8)
Naruse N [35]	2022	Japan	Retrospective	Various	42	97.6%	88.1%	4 (9.5)

SEMS, self-expandable metal stent; N, number.

## Data Availability

Data sharing is not applicable.
